# A randomized controlled trial on the effect of hydroxychloroquine in mild Graves’ orbitopathy (GO-HCQ): study protocol

**DOI:** 10.1186/s13063-025-09002-6

**Published:** 2025-08-04

**Authors:** Chia-Hung Lin, Yi-Hsuan Wei, Jin-Ying Lu, Hung-Yuan Li, Chung-Wei Lee, Chung-Yi Yang, Chin-Hao Chang, Wan-Chen Wu, Chih-Yuan Wang, Shyang-Rong Shih

**Affiliations:** 1 Division of Endocrinology and Metabolism, Department of Internal Medicine, National Taiwan University Hospital, Taipei, Taiwan; 2 Department of Ophthalmology, National Taiwan University Hospital, Taipei, Taiwan; 3 Department of Medical Imaging, National Taiwan University Hospital, Taipei, Taiwan; 4 Department of Medical Imaging, I-Shou University, E-Da Hospital, Kaohsiung, Taiwan; 5 Department of Medical Research, National Taiwan University Hospital, Taipei, Taiwan; 6 Department of Internal Medicine, National Taiwan University College of Medicine, No. 1, Section 1, Ren-Ai Road, Taipei, 10051 Taiwan

**Keywords:** Graves’ orbitopathy, Thyroid eye disease, Graves’ disease, Hydroxychloroquine

## Abstract

**Background:**

Graves’ orbitopathy (GO) is a prevalent manifestation of Graves’ disease (GD), characterized by proptosis, eyelid retraction, soft tissue swelling, diplopia, and potential visual acuity impairment. Furthermore, even mild GO can significantly impact mental health and overall quality of life for patients with GD. Despite its severity, available medical treatments for mild GO are limited. Recent basic studies on orbital fibroblasts suggest hydroxychloroquine (HCQ) as a promising therapeutic agent for GO patients. This randomized controlled trial (RCT) was designed to assess the efficacy of HCQ in treating mild GO.

**Methods:**

This multi-center open-label RCT will be conducted in Taiwan with a total of 108 participants randomized into HCQ and control groups at 2:1 allocation ratio. The primary endpoint of this study is a composite outcome of ophthalmic parameters including eyelid aperture, soft tissue involvement, and exophthalmos. Secondary endpoints comprise changes in quality of life (GO-QoL), orbital volumetry via computed tomography (CT), diplopia scores, clinical activity scores (CAS), visual acuity, and thyroid autoantibodies.

**Discussion:**

This RCT will elucidate the clinical benefits of oral HCQ in patients with mild GO, assessing ophthalmic outcomes, quality of life, disease activity, and thyroid autoantibodies. In addition, data obtained from orbital CT measurements will provide valuable insights into subtle changes in orbital fat and extra-ocular muscle volumes, potentially offering an objective tool for monitoring GO progression.

**Trial registration {2a, 2b}:**

ClinicalTrials.gov NCT05126147. Registered on November 2021. https://clinicaltrials.gov/study/NCT05126147. All items from the World Health Organization (WHO) Trial Registration Data Set are addressed within the relevant sections of this protocol.

**Supplementary Information:**

The online version contains supplementary material available at 10.1186/s13063-025-09002-6.

## Introduction

### Background and rationale {6a}

Approximately 20% of patients with Graves’ disease (GD) suffer from Graves’ orbitopathy (GO). The central pathogenic mechanism of GD is the TSH receptor antibody which causes hyperthyroidism and triggers autoimmune inflammation in the eyes, leading to thickening of the extra-ocular muscles, increased fat deposition, and the accumulation of hyaluronic acid and glycosaminoglycans (GAGs) [1]. These manifestations constitute the typical symptoms of GO, such as swollen and inflamed eyes, exophthalmos, eyelid swelling, diplopia, and even impaired vision. In addition to the physical discomfort, the unpleasant appearance of the eyes caused by GO severely affects patients’ self-confidence, resulting in psychological and social barriers [2]. 

Only half of patients with mild GO naturally improve, and about 20% of them may worsen during follow-up [3]. Unfortunately, there are limited treatment options for patients with mild GO, and the only suggested medical treatment for mild GO is selenium [4]. However, the supporting evidence of selenium in treating mild GO was conducted in the selenium-deficient Europe. Whether selenium is effective in selenium-sufficient region including Taiwan remains unknown [5]. In addition, literature revealed potential association between excessive selenium and certain malignancies and type 2 diabetes mellitus [6], which makes selenium supplementation in selenium-sufficient area more controversial.

In contrast to mild GO, the treatment of sight-threatening or moderate-to-severe GO is more established, with glucocorticoid steroid or orbital surgery as the mainstream strategy, which are often not suitable for mild GO patients [7]. Furthermore, emerging therapies including mycophenolate sodium [8] and teprotumumab [9] focused only on moderate-to-severe and active GO. As a result, a significant proportion of patients with mild GO experience decrease in their quality of life, and there is an unmet need that no effective treatment options are available.

Hydroxychloroquine (HCQ) is a less toxic metabolite of chloroquine and has been increasingly used for long-term and chronic treatment of autoimmune diseases owing to its immune-modulatory effects [10]. One cell study published in *Journal of Clinical Endocrinology and Metabolism* investigated the effect of HCQ on the orbital fibroblasts from 10 patients with mostly mild and inactive GO [2]. Results revealed that HCQ significantly inhibited proliferation, mitotic clonal expansion, adipogenesis, and hyaluronic acid on orbital fibroblasts. However, the evidence of clinical use of HCQ for GO is lacking. Thus, we designed an open-label randomized controlled trial to examine the effect of HCQ in patients with mild GO (NCT05126147).

### Objectives {7}

The objective of this study is to compare the use of oral HCQ versus observation on the ophthalmic outcomes in patients with mild GO. In addition to ophthalmic measurements, quality of life, orbital computed tomography (CT), diplopia score, clinical activity score (CAS), visual acuity, and thyroid autoantibodies including anti-thyroid peroxidase antibodies (anti-TPO), anti-thyroglobulin antibody (ATA), and thyrotropin-binding inhibitory immunoglobulins (TBII) will be recorded for comparison.

### Trial design {8}

This is a multi-center open-label interventional randomized controlled trial (RCT) comparing the effects of HCQ versus observation in patients with mild GO in Taiwan. The allocation ratio of randomization will be 2:1 in HCQ and control group, respectively. This trial adopts a superiority framework, hypothesizing that the addition of hydroxychloroquine to standard of care will result in superior ophthalmic outcomes compared to standard of care alone in patients with mild GO.

## Methods: participants, interventions, and outcomes

### Study setting {9}

Patients with mild GO who have already achieved euthyroidism will be screened at Taipei, Bei-Hu, Cancer Center, and Hsin-Chu branches of National Taiwan University Hospital (NCT05126147). Participants will be enrolled and randomly assigned in a 2:1 ratio to treatment or control group. The treatment group will receive HCQ 200 mg twice daily for a duration of 6 months, followed by a 6-month observation period, while the control group will receive standard of care for GO by endocrinologists. Participants will be enrolled by endocrinologists after screening of the inclusion and exclusion criteria. Ophthalmic measurements will be performed by the same ophthalmologist specializing in GO in each hospital to avoid inter-observer differences. In addition, ophthalmologists will be blinded to the participants’ treatment groups. They will be excluded from any role in allocation or treatment decisions and conducted evaluations according to standardized procedures.

### Eligibility criteria {10}

We estimate enrolling 108 patients with mild GO meeting the following inclusion and exclusion criteria.

#### Inclusion criteria

1. Participants aged 18 to 75 years.
2. The diagnosis of mild GO established by an endocrinologist and an ophthalmologist based on clinical symptoms, signs, and laboratory data, according to the European Group on Graves’ orbitopathy (EUGOGO) criteria.
3. Participants should achieve euthyroidism for at least 2 months. If they have undergone radioactive iodine treatment due to hyperthyroidism, their thyroid function should be normal for at least 6 months.

#### Exclusion criteria

1. Participants with moderate-to-severe or sight-threatening GO
2. Pregnancy
3. Drug or alcohol abuse
4. Inability to comply with the trial requirements
5. Inability to provide informed consent
6. Administration of HCQ or systemic steroids within 3 months prior to enrollment
7. History of adverse effects related to HCQ such as retinopathy
8. Pre-existing retinal disease, including macular pathology (e.g., age-related macular degeneration or diabetic macular edema)
9. Renal insufficiency (estimated glomerular filtration rate (eGFR) less than 60 mL/min)
10.  Abnormal liver function (aspartate aminotransferase (AST) or alanine aminotransferase (ALT) more than 2 times the upper limit)
11.  Anemia (hemoglobin less than 10 g/dL)
12.  Granulocyte deficiency (absolute neutrophil count less than 100/µL)
13.  Low platelet count (platelet less than 150 K/µL)
14.  G6PD (glucose-6-phosphate dehydrogenase) deficiency
15.  Porphyria cutanea tarda
16.  Allergy to 4-aminoquinoline compounds

### Who will take informed consent? {26a}

The participants will be required to sign formal informed consents after a full explanation of the details regarding this trial by principal investigators and/or study nurses.

### Additional consent provisions for collection and use of participant data and biological specimens {26b}

Permission of use of personal data, medical record, and biological specimens will be obtained in the formal informed consents.

## Interventions

### Explanation for the choice of comparators {6b}

According to previous epidemiological evidence, around half of the patients with mild GO experience remission spontaneously [6]. Watchful waiting is also recommended as one of the treatment options in the guidelines by EUGOGO, American Thyroid Association (ATA), and European Thyroid Association (ETA) [7, 11]. Selenium was reported to be effective in Europe in one previous study [4], but the effect of selenium on GO in selenium-sufficient region including Taiwan remains unknown [5]. Therefore, we chose observation and standardized care for GO as the comparator in the control group. The standard of care consists of supportive treatment as recommended by EUGOGO guidelines [7], including regular monitoring, local symptomatic relief (e.g., lubricating eye drops), optimization of thyroid function, and lifestyle counseling such as smoking cessation. These approaches represent the current clinical management for patients with mild GO who do not meet criteria for corticosteroid therapy.

### Intervention description {11a}

Treatment group will receive HCQ 200 mg twice daily for 6 months. The treatment dosage is commonly used clinically as an initial starting dose, with average serum concentration of 0.3 to 1.9 μM in steady state [12]. The effect of HCQ was supported by one previous cell study of orbital fibroblast derived from patients with GO treated with physiological achievable concentration of HCQ [2]. Both the intervention and control groups will receive the standard of care.

### Criteria for discontinuing or modifying allocated interventions {11b}

Trial discontinuation will occur under the following conditions for enrolled participants:

1. Disease deterioration during study period requiring further medical treatment or surgical interventions
2. Intolerance, documented adverse events, or allergy to study drug (i.e., HCQ) in treatment group

### Strategies to improve adherence to interventions {11c}

Principal investigator and co-investigators will explain the whole study protocol and meanings of randomization to the participants before enrollment to make sure they understand and are able to comply with the study schedule. After randomization, study nurses will maintain close contact with participants and ask them to report adverse events and return for examinations and outpatient clinic visits.

### Relevant concomitant care permitted or prohibited during the trial {11d}

Standardized care for patients with GO will be provided in every enrolled subject, including treatment of hyperthyroidism to achieve euthyroidism, refrain from smoking, or use of eyedrops or lubricants for dry eye. During study period, additional use of systemic steroid (oral or intravenous) is prohibited.

### Provisions for post-trial care {30}

Immediate consultation and medical assistance will be provided to participants with suspected adverse events related to HCQ without additional expenses. After trial completion, the patients will be followed regularly by their endocrinologist and ophthalmologist to discuss further treatment strategies for GO.

### Outcomes {12}

Primary endpoint of this study is the composite ophthalmic outcome of ophthalmic examinations including eyelid aperture, soft tissue involvement, and exophthalmos at the 24th week and 48th week of study, with definitions in line with the previous literature for comparison [4]. 

The primary endpoint is a composite ophthalmic outcome assessed at weeks 24 and 48. Based on predefined criteria (as shown in Table 1), participants will be classified as follows:

- Improvement: achievement of at least one improvement criterion in any domain, without meeting any deterioration criteria in the others
- Deterioration: meeting any deterioration criterion in one or more domains
- Stable: neither improved nor deteriorated in any domain

**Table 1:** Definition of the composite ophthalmic outcome (primary endpoint)

Ophthalmic domain	Measure	Result classifications		
		Improvement	Stable	Deterioration
Eyelid aperture	Slit lamp	Decrease by ≥ 2 mm	Neither improved nor deteriorated	–
Soft tissue involvement	NOSPECS class 2 signs	Reduction of ≥ 1 grade in NOSPECS class 2 signs		1. Increase ≥ 1 NOSPECS class 2. New NOSPECS class
Exophthalmos	Hertel exophthalmometer	Decrease by ≥ 2 mm		–
Other ophthalmic signs				Vision acuity deterioration or other evidence of optic nerve compression

NOSPECS is a clinical classification system for assessing the severity of Graves’'orbitopathy [7], consisting of the following seven classes (class 0–6):

Class 0: No signs or symptoms; Class 1: Only signs, no symptoms (e.g., lid retraction); Class 2: Soft tissue involvement (e.g., periorbital edema, conjunctival injection, chemosis); Class 3: Proptosis; Class 4: Extraocular muscle involvement; Class 5: Corneal involvement; Class 6: Sight loss due to optic nerve compression

In this study, improvement or deterioration in soft tissue involvement is assessed based on changes in class 2 severity. Deterioration is defined as a worsening of at least one grade in any NOSPECS class, or the appearance of a new class not previously present Improvement: achievement of at least one improvement criterion in any domain, without meeting any deterioration criteria in the others Deterioration: meeting any deterioration criterion in one or more domains Stable: neither improved nor deteriorated in any domain

Secondary endpoints:

1. Changes in GO-Quality of Life Questionnaire (GO-QoL) scores (visual functioning and appearance subscales) at the 24th week and 48th week: A change of 6 points is considered a minimal clinically important difference (MCID). An increase of 6 points in any category is considered improvement, a decrease of 6 points is considered deterioration, and anything in between is classified as stable [13].
2. Changes in non-contrast orbital CT at the 24th week and 48th week, including the ratio and density differences of muscle volume, fat volume, and orbital volume [14].
3. Diplopia will be assessed at the 24th and 48th weeks using the Gorman score, a validated grading system endorsed by the EUGOGO [15]. This scale classifies diplopia into four categories:
  1. 0—no diplopia
  2. 1—intermittent diplopia
  3. 2—inconstant diplopia
  4. 3—constant diplopia in the primary position or on reading
4. CAS, measured by the 7-point CAS [16], at the 24th week and 48th week.
5. Visual acuity, assessed using best-corrected visual acuity (BCVA, Snellen chart), at the 24th week and 48th week.
6. Changes in serum inflammatory and fibrosis markers at the 24th week and 48th week.
7. Changes in thyroid antibodies at the 24th week and 48th week.

### Participant timeline {13}

Time schedule of enrollment, interventions, and assessments is illustrated in Table 2.

**Table 2:**
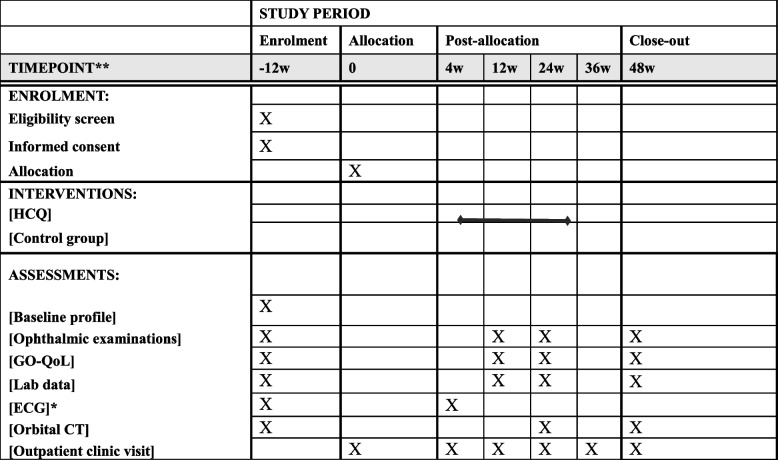
Time schedule of enrolment, interventions, and assessments

*CT* computed tomography, *ECG *electrocardiogram, *GO-QoL* Graves' ophthalmopathy quality of life questionnaire, *HCQ* hydroxychloroquine. * *ECG* was performed in treatment group to rule out *HCQ*-related *QT* prolongation

### Sample size {14}

The sample size was estimated using Stata software. Due to the lack of literature on the effects of HCQ for mild GO, we referred to the clinical trial published by the NEJM in 2011, which investigated the efficacy of selenium in patients with mild GO [4]. Approximately 12% of GO patients showed spontaneous improvement, and the use of selenium significantly increased this improvement rate to 37% (absolute difference of 25%). We hypothesized that HCQ improves the prognosis of mild GO patients by at least the same proportion as selenium, i.e., 25% of improvement rate [3]. As selenium is now considered a recommended treatment option for mild GO in clinical guidelines, we reasoned that hydroxychloroquine should demonstrate at least comparable efficacy to warrant further investigation. Based on a 2:1 random allocation ratio, a two-sided *α* of 0.05, and 80% power, the estimated sample size was 72 participants in the HCQ group and 36 in the control group.

### Recruitment {15}

We will collaborate with endocrinologists and ophthalmologists at the Taipei, Bei-Hu, Cancer Center, and Hsin-Chu branches of National Taiwan University Hospital to screen potential candidates eligible for the study.

### Assignment of interventions: allocation {16a, 16b, 16c}

A total of 108 participants will be randomly assigned into experimental group and control group with a 2:1 allocation. The randomization process will be independently constructed by the National Taiwan University Hospital Statistical Consulting Unit (NTUH-SCU). Randomization sequence will be created using SAS 9.4 (SAS Institute Inc, Cary, NC). The result of randomization will be delivered to principal investigators via Research Electronic Data Capture (REDCap) system, version 14.3.3, a secure web-based application by Vanderbilt University.

### Assignment of interventions: blinding {17a, 17b}

This study is an open-label study, so all participants and treating physicians will be unblinded. To minimize detection bias, ophthalmologists performing outcome assessments will be blinded to group allocation. They have no role in randomization or participant assignment and will perform standardized evaluations without access to treatment status.

### Data collection and management {18a, 19}

Study nurses will make appointments with patients to ensure essential data are collected completely, including baseline information, lab data, GO-QoL, orbital CT, and ophthalmic examinations according to study schedule (Table 2). All clinical data will be recorded in standardized case report forms (CRFs) by trained study nurses at each study site. After data collection, information will be stored electronically in either the electronic medical record system or the REDCap platform, a centralized, password-protected database accessible only through the internal hospital network to ensure privacy and confidentiality. All data entries will be validated through regular data audits, range checks, and periodic double-entry verification for critical variables. Access to the database will be restricted to authorized personnel only. Data will be anonymized using participant identification codes. The database is hosted on a secure institutional server with regular backups and encryption protocols to ensure data integrity and confidentiality. Detailed data collection in this study is shown as below:

1. Basic information: At the time of enrollment, we will collect participants’ age, gender, nationality, smoking status or exposure to secondhand smoke, types of thyroid disease, treatments received for thyroid disease in the past, current medications, duration of eye symptoms, and autoimmune diseases. Additionally, it is necessary to inquire if the patient is pregnant or planning to become pregnant, and a urine pregnancy test may be conducted if needed to rule out the possibility of pregnancy.
2. GO-QoL will be obtained at enrollment and at the 12th, 24th, and 48th weeks. This internationally recognized questionnaire, validated for both quantification and validity, will be administered by study nurses [13]. It is designed to assess the quality of life in patients with GO, focusing on two main aspects: visual functioning and appearance subscales. Scores for each aspect are calculated separately, with a maximum score of 100 points each; lower scores indicate a greater impact on quality of life. This questionnaire has been translated into traditional Chinese and used in prior research and published internationally [17].
3. Ophthalmological examinations will be conducted by the same ophthalmologist in each hospital. Three main metrics will be measured: eyelid aperture, soft tissue involvement, and exophthalmos using a Hertel exophthalmometer. These metrics are the primary outcomes of the study. Other assessments, including diplopia evaluated using the Gorman score [15], clinical activity score, and visual acuity, will also be recorded in detail. Given the potential side effect of hydroxychloroquine causing retinopathy [18], ophthalmologists will perform baseline and follow-up retinal toxicity assessments, including optical coherence tomography (OCT) and automated visual field testing when indicated.
4. Orbital non-contrast CT will be conducted to evaluate changes in CT measurement values before enrollment, after medication, and after discontinuation of medication. Measurements for orbital volumetry will be based on a unified method adopted in numerous studies [14]. Parameters will include muscle volume (MV), fat volume (FV), orbital volume (OV), MV/OV ratio, FV/OV ratio, exophthalmos length (distance from interzygomatic line to the apex of the cornea), mean density of muscle, and mean density of fat.

### Collection, laboratory evaluation, and storage of biological specimens {33}

Blood specimens will be collected via venipuncture, then centrifuged and aliquoted into Eppendorf tubes, and stored at − 80 °C. Laboratory evaluations will include thyroid function tests (free thyroxine [free T4], thyroid-stimulating hormone [TSH]), thyroid autoantibodies (TBII, anti-thyroid peroxidase [anti-TPO], and anti-thyroglobulin antibody [ATA]), complete blood count with differential, liver function tests (AST, ALT), inflammatory markers (interleukin-6 [IL-6], interleukin-1β [IL-1β], tumor necrosis factor-α [TNF-α], and interferon-γ [IFN-γ]), fibrosis marker (transforming growth factor-β [TGF-β]), and serum hyaluronic acid levels. Additional serum and plasma samples will be stored at − 80 °C for up to 20 years for future research related to autoimmune or thyroid-related diseases, in accordance with participants’ informed consent and institutional ethics approval. All remaining biospecimens will be destroyed at the end of the storage period in compliance with institutional biosafety protocols.

### Statistical methods {20a, 20b, 20c}

Basic characteristics will be presented using descriptive statistics, including mean (standard deviation) and number of occurrences (percentage). For non-normally distributed data, the median (interquartile range, IQR) will be used. To compare between the treatment group and the observation group, depending on the nature of the data, Student’s *t*-tests will be used for continuous variables, and chi-square tests or Fisher’s exact tests will be used for categorical variables, to examine the differences between the two groups.

To compare the scores of the GO-QoL and the CAS before and after treatment, two-sided *t*-tests and Mann–Whitney tests will be used. Pearson’s correlation analysis will be utilized to test the association and determine the correlation coefficient (*r*) among variables of interest. The severity of thyroid eye disease before and after treatment in both groups will be compared in terms of the proportion of patients who show improvement, stability, or deterioration. One-way repeated measures analysis of variance (ANOVA) will be used to analyze the change of variables before and after treatment. In addition to ANOVA, ANCOVA and regression models will be used to adjust for baseline differences and estimate effect sizes with confidence intervals.

Exploratory subgroup analyses will be performed to assess whether treatment effects differ by age, gender, baseline TBII levels, duration of GO, and smoking status. These analyses are intended to explore potential effect modifiers and will be interpreted cautiously given the limited sample size. A two-tailed *P* value < 0.05 will be considered statistically significant. Statistical analysis will be performed using Stata/SE 14.0 for Windows (StataCorp, College Station, TX, USA).

All randomized participants, including those who discontinue the intervention prematurely, will be included in the primary analysis based on the intention-to-treat (ITT) principle. Sensitivity analyses will be conducted if necessary to evaluate the robustness of results in the presence of missing data. Missing data will be handled using multiple imputation under the assumption of missing at random (MAR).

### Interim analyses {21b}

No formal interim analysis is planned due to the small sample size and short treatment duration. The study is not designed for early stopping based on efficacy or futility. Adverse events will be monitored continuously throughout the trial by the study investigators.

### Plans to give access to the full protocol, participant-level data, and statistical code {31c}

Data collected in this study will not be publicly accessible but could be made available upon request for academic purposes after de-identification.

### Oversight and monitoring {5d, 21a, 23}

The core members of this trial committee, including clinical trial experts, statisticians, and ethical advisors from the National Taiwan University Hospital Clinical Trial Center (NTUH-CTC), will provide advisory support for developing the trial protocol, monitoring data, supervising safety, and reporting adverse events. Periodic research audits will be carried out to ensure the trial’s adherence to the predetermined protocol and active progression. Ethical considerations and modifications to the study protocols will be overseen by the National Taiwan University Hospital Research Ethics Committee.

### Adverse event reporting and harms {22}

All adverse events (AEs) and serious adverse events (SAEs) will be monitored and recorded throughout the study period. Known potential adverse reactions to hydroxychloroquine, including rash, gastrointestinal upset, headache, and visual symptoms, will be specifically inquired about at each ophthalmologic and clinical follow-up visit. Unexpected adverse events will also be collected and documented. All reported events will be coded using the Medical Dictionary for Regulatory Activities (MedDRA) terminology where applicable. Both expected and unexpected adverse events will be included in the trial publications, following the Consolidated Standards of Reporting Trials (CONSORT) extension for harms reporting guidelines.

### Protocol amendments {25}

Any modifications to the protocol that may impact study conduct, participant safety, or study outcomes—including changes to eligibility criteria, outcomes, analyses, or study procedures—will be submitted to the Research Ethics Committee (REC) in National Taiwan University Hospital for review and approval prior to implementation. Substantial protocol amendments will also be updated in the ClinicalTrials.gov registration and communicated to investigators and participants as appropriate. All amendments will be reported in future trial publications.

## Discussion

There have been unmet medical needs for patients with mild GO, given that they still suffer from overt decline of quality of life [13, 19]. Despite the discovery in 2011 that selenium supplementation could benefit patients with mild GO, documented by a RCT published in the *New England Journal of Medicine* [4], there are no other effective medications emerged since then. In addition, the effect of selenium in selenium-sufficient region remains questionable to date [7]. Furthermore, the exploration of other potential treatments for mild GO has been limited, with few clinical trials registered or ongoing on ClinicalTrials.gov.

In 2020, one cell study published in the *Journal of Clinical Endocrinology and Metabolism* showed the effects of HCQ on orbital fibroblasts [2]. It disclosed new possibilities on this challenging disease, and this pivotal finding has motivated the initiation of our clinical trial to ascertain whether HCQ’s effects could be replicated in patients with mild GO.

In this open-label study, we acknowledge potential biases such as performance and detection bias. To mitigate these, we will ensure that all participants receive the same standard of care in accordance with EUGOGO guidelines. Ophthalmologists performing outcome assessments will be blinded to group allocation and will not be involved in participant randomization or treatment delivery. They will conduct standardized assessments independently to reduce subjective bias. Additionally, the study will utilize randomized participant selection and objective outcome measures such as ophthalmic outcome and orbital CT parameters to further reduce the risks of selection and reporting biases.

Several challenges may be encountered in the conduct of this clinical trial. For example, the randomization process makes some patients felt uncertain, especially in those who are eager to receive effective medications, which can be overcome through further explanation of the scientific rationale for randomization. In addition, concerns about the potential side effects of HCQ, such as retinopathy, have been raised in the literature. This has contributed to hesitancy in joining the study, particularly among patients with mild GO whose quality of life is only minimally affected. These questions will be addressed by providing detailed information about the likelihood of such effects and the monitoring strategies in place. Finally, potential operational challenges, such as scheduling or communication difficulties, have been anticipated and addressed through flexible coordination strategies, including proactive use of social messaging platforms, appointment reminders, and individualized rescheduling when necessary. 

In conclusion, this protocol outlines a randomized controlled trial designed to address the unmet therapeutic needs of patients with mild GO. By anticipating potential recruitment, operational, and compliance challenges, we have incorporated adaptive strategies and resource planning to support trial implementation. We expect that the results of this study will contribute to the development of novel treatment strategies for patients with mild GO. 

## Supplementary Information

Additional file 1: SPIRIT checklist

Additional file 2: Informed consent form

## Data Availability

Data collected in this study will not be publicly accessible but could be made available upon request for academic purposes after de-identification.

## Abbreviations

ALT: Alanine aminotransferase
ANOVA: Analysis of variance
AST: Aspartate aminotransferase
ATA: American Thyroid Association
anti-TPO: Anti-thyroid peroxidase antibodies
ATA: Anti-thyroglobulin antibody
CAS: Clinical activity score
CT: Computed tomography
DMC: Data monitoring committee
eGFR: Estimated glomerular filtration rate
ETA: European Thyroid Association
EUGOGO: European Group on Graves’ orbitopathy
free T4: Free thyroxine
FV: Fat volume
G6PD: Glucose-6-phosphate dehydrogenase
GAGs: Glycosaminoglycans
GD: Graves’ disease
GO: Graves’ orbitopathy
GO-QoL: GO-Quality of Life Questionnaire
HCQ: Hydroxychloroquine
IFN-γ: Interferon γ
IL-1β: Interleukin 1β
IL-6: Interleukin 6
MCID: Minimal clinically important difference
MV: Muscle volume
NTUH-SCU: National Taiwan University Hospital Statistical Consulting Unit
NTUH-CTC: National Taiwan University Hospital Clinical Trial Center
OV: Orbital volume
RCT: Randomized controlled trial
TBII: Thyrotropin-binding inhibitory immunoglobulins
TGF-β: Transforming growth factor β
TNF-α: Tumor necrosis factor α
TSH: Thyroid-stimulating hormone
