# Supplementary material for: A randomized controlled trial on the effect of hydroxychloroquine in mild Graves’ orbitopathy (GO-HCQ): study protocol
Source: Trials. 2025 Aug 4;26:272. doi: 10.1186/s13063-025-09002-6 (PMC12323228; doi:10.1186/s13063-025-09002-6)
Supplement: Supplementary file 2 — Additional file 2: Informed consent form [file 13063_2025_9002_MOESM2_ESM.doc]

| 計畫名稱  中文：以hydroxychloroquine治療甲狀腺眼病變之隨機對照臨床試驗  英文: A randomized controlled trial on the effect of hydroxychloroquine in Graves’ orbitopathy. | |
| --- | --- |
| 試驗機構：  國立台灣大學醫學院附設醫院  內科部 | 委託單位/藥廠：無  研究經費來源：院內計畫或科技部 |
| 試驗主持人：施翔蓉 職稱：台大醫院內科部副教授、主治醫師  協同主持人：楊偉勛 職稱：台大醫院內科部教授、主治醫師  廖述朗 職稱：台大醫院眼科部教授、主治醫師  呂金盈 職稱：台大醫院內科部主治醫師  李弘元 職稱：台大醫院內科部副教授、主治醫師  吳婉禎 職稱：台大醫院內科部講師、主治醫師  魏以宣 職稱：台大醫院眼科部講師、主治醫師  李崇維 職稱：台大醫院影像醫學部助理教授、主治醫師  林家宏 職稱：台大新竹分院內科部主治醫師  粘峯榕 職稱：台大癌醫中心分院內科部主治醫師  林彥博 職稱：台大北護分院內科部主治醫師 | |
| 聯絡人：施翔蓉 上班時間聯絡電話：(02)23123456分機261613 | |
| 受試者姓名： | 病歷號碼： |
| 您被邀請參與此臨床試驗/研究，這份表格提供您本試驗/研究之相關資訊，試驗主持人或其授權人員將會為您說明試驗/研究內容並回答您的任何疑問，在您的問題尚未獲得滿意的答覆之前，請不要簽署此同意書。您不須立即決定是否參加本試驗/研究，請您經過慎重考慮後方予簽名。您須簽署同意書後才能參與本試驗/研究。如果您願意參與本試驗/研究，此文件將視為您的同意紀錄。即使在您同意後，您仍然可以隨時退出本試驗/研究而不需理由。 | |
| 1. 試驗/研究目的：   甲狀腺眼病變是一種常見發生在葛瑞夫氏甲狀腺亢進病患的一種眼睛表現，但對於輕度甲狀腺眼病變者大多僅有建議觀察，除了補充硒之外沒有其他藥物建議。近期研究顯示hydroxychloroquine在細胞實驗上有潛在好處。本試驗目的在於研究使用免疫調節製劑hydroxychloroquine治療甲狀腺眼病變，觀察對於眼科表現、生活品質、電腦斷層上之參數變化、血中生物指標、血中纖維細胞(fibrocyte)等是否有所影響。 | |
| 1. 研究背景或藥品/醫療技術/醫療器材現況：   羥氯奎寧(hydroxychloroquine, Plaquenil®)原為預防或治療瘧疾之用藥，現今亦作為免疫調節劑，已取得美國藥品食品管理局與台灣衛生福利部的適應症，可用於多種自體免疫疾病如：全身性紅斑性狼瘡，風濕性關節炎等的治療，但適應症尚未包含甲狀腺眼病變。 | |
| 1. 試驗/研究之納入與排除條件：   執行本研究計畫的醫師或相關研究人員將會與您討論有關參加本研究的必要條件。請您配合必須誠實告知我們您過去的健康情形，若您有不符合參加本研究的情況，將不能參加本研究計畫。   - 1. 符合下列條件者，適合參加本試驗：經代謝內分泌科醫師診斷之輕度甲狀腺眼病變的患者(18歲至75歲)，甲狀腺功能正常，除了眼藥水外未曾接受過任何針對甲狀腺眼病變之治療。   2. 若有下列情況者，不能參加本試驗：      1. 影響視力之甲狀腺眼病變      2. 已懷孕或是計畫懷孕之成年女性      3. 藥物或酒精濫用、無法遵從試驗      4. 無法取得知情同意書      5. 收案前三個月使用過hydroxychloroquine或全身性類固醇      6. 過去曾發生或hydroxychloroquine引起之副作用如視網膜病變      7. 本身已有視網膜病變者      8. 腎功能不全者 (estimated CCr < 60ml/min)      9. 肝功能異常者 (AST or ALT > 2 x upper limit)      10. 貧血 (Hb < 10g/dL)      11. 顆粒性白血球缺乏症 (absolute neutrophil count < 100/uL)      12. 血小板過低 (PLT < 150K/uL)      13. 葡萄糖-6-磷酸脫氫酶缺乏症 (G6PD deficiency)，俗稱蠶豆症      14. 緩發性皮膚病變紫質症 (porphyria cutaneous tarda)      15. 對4-aminoquinoline化合物過敏者。   3. 總預定收納人數：108人。 | |
| 1. 本試驗/研究方法及相關程序：    1. 首先，確認您符合收案資格後，我們會進行基本資料詢問、問卷調查（生活品質評估，將花費約10分鐘）、眼科檢查、電腦斷層及抽血檢查。    2. 我們將以「隨機分配」分配您進入之組別，這代表您是按照機率（如同擲銅板一般）進入其中一組。您被分配到實驗組的機率是三分之二、對照組的機率是三分之一。       1. 若您歸類於實驗組，我們會開立口服標準劑量的免疫調節製劑：羥氯奎寧(hydroxychloroquine)/必賴克廔(Plaquenil®)一顆(200毫克)早晚各一次，給您開始服用來治療甲狀腺眼病變。       2. 若您歸類於對照組，則根據常規方式持續治療甲狀腺眼病變。    3. 兩組均於研究開始後第三個月及第六個月用藥期滿後回診追蹤抽血與眼科檢查。第六個月時也會接受問卷調查及電腦斷層檢查。第十二個月時會再進行抽血檢查、眼科檢查及電腦斷層檢查。    4. 整個研究過程中抽血一共四次，分別於收案時、第三個月、第六個月及第十二個月。每次抽血量約為10cc左右，檢驗項目包括甲狀腺功能、全血球計數、肝功能、甲狀腺抗體、血中發炎指標、纖維化指標、玻尿酸濃度等。    5. 追蹤期間，如有任何不適，請提前回診。在試驗期間如果發生任何與本藥物(羥氯奎寧)相關之副作用，則停止本藥物治療，進行標準常規治療。    6. 試驗時間總計為12個月，用藥時間6個月，持續觀察時間6個月。    7. 總共收納試驗人數為108人。 | |
| 1. 可能發生之風險及其發生率與處理方法：    1. 與試驗藥物/醫療器材/醫療技術相關的風險 (本試驗使用藥物/器材/醫療技術的副作用)： (1) 採集血液樣本：從手臂上抽血可能會引起部位疼痛、瘀青、頭昏眼花，而在很低的機率下可能會發生感染。處理方式為抽血後需按壓抽血部位至少5分鐘；瘀青可以熱敷方式緩解；頭昏眼花則需靜坐或平躺休息。空腹可能引起頭暈、頭痛、胃不舒服或暈倒。處理方式為靜坐休息，抽血後盡快進食。以上副作用均為輕微程度。 (2) 藥物相關風險：羥氯奎寧(hydroxychloroquine)如未超過每日建議劑量(每日400毫克)、使用時間未達5年以上，則發生視網膜病變之嚴重副作用的機率極低。在開始接受藥物治療前會安排您先至本院眼科檢查，排除上述疾病後，才開始藥物治療。同時我們會告知您如在服用期間發生任何視力異常，包括色彩視覺異常或是視野缺損，則立即停用藥物，並且接受持續性的觀察與眼科檢查。   曾有發生皮膚發疹、搔癢、皮膚及黏膜色素改變的個案報告，一般而言，停藥後這些輕微副作用均可迅速改善。  少數人會產生腸胃道不適，如：噁心、下痢、腹痛等，這些輕微副作用可在劑量減低或停藥後立即緩解。  除此之外，文獻中有顯示該藥物可能造成心電圖QTc延長，因此在開始接受藥物治療前若無心電圖資料會安排心電圖檢查，使用藥物後也會追蹤心電圖檢查，排除此情況後才繼續用藥。  註：羥氯奎寧(hydroxychloroquine)最大劑量為800毫克   - 1. 與試驗/研究過程相關的風險：   受試者會於收案時、第六個月及第十二個月時接受眼眶電腦斷層檢查，因此將暴露輻射線，單次電腦斷層輻射量約為0.86毫西弗（mSv)，執行三次預估約為2.58毫西弗。根據行政院原子能委員會資料，台灣每人每年接受的天然背景輻射劑量約為1.62毫西弗。兩者合併預估輻射暴露量為4.2毫西弗，相當於210張一般胸部X光片，然而該劑量並未超過國際放射防護委員會所建議之個人安全劑量上限5毫西弗。  如有任何研究過程或藥物造成之副作用，請立即與本研究主持人施翔蓉醫師聯絡，國立臺灣大學醫學院附設醫院將提供您必要之醫療照護。 | |
| 1. 其他替代療法及說明：   您不一定需要參加本試驗，您可在門診持續追蹤甲狀腺功能與甲狀腺眼病變之情況，如有進展至嚴重之甲狀腺眼病變，則考慮給予類固醇治療或進行手術。 | |
| 1. 試驗/研究預期效益：   目前國內外均無任何相同的臨床試驗的文獻報告。就學理上而言，預期羥氯奎寧的治療可能可以降低甲狀腺眼病變之凸眼程度及生活品質等。 | |
| 1. 試驗/研究進行中受試者之禁忌、限制與應配合之事項：   當您參加本試驗期間，為了您的安全，請您配合以下事項：   - 1. 避免富含碘的食物，如：紫菜與海帶；避免接受含碘之藥物(如：抗心律不整藥物-臟得樂)治療或需要打顯影劑的檢查   2. 育齡期的成年女性請注意避孕，有效之避孕方式如：口服避孕藥、荷爾蒙注射、保險套或植入避孕器。若您於試驗間，有可能懷孕的疑慮，請立即告知試驗醫師。   3. 避免抽菸或暴露於二手菸之環境，以防止甲狀腺眼病變之加重。   4. 提供您的過去病史、醫療紀錄及和目前病情有關的正確資訊   5. 為了您的安全，請告知試驗醫師您出現的任何不舒服症狀。   6. 若您有任何疑問，請不要客氣，請和您的試驗人員(醫師、護理師)直接提出。 | |
| 1. 受試者個人資料之保密：   台大醫院將依法把任何可辨識您的身分之記錄與您的個人隱私資料視為機密來處理，不會公開。研究人員將以一個研究代碼代表您的身分，此代碼不會顯示您的姓名、國民身分證統一編號、住址等可識別資料。如果發表試驗/研究結果，您的身分仍將保密。您亦瞭解若簽署同意書即同意您的原始醫療紀錄可直接受監測者、稽核者、研究倫理委員會及主管機關檢閱，以確保臨床試驗/研究過程與數據符合相關法律及法規要求，上述人員並承諾絕不違反您的身分之機密性。除了上述機構依法有權檢視外，我們會小心維護您的隱私。 | |
| 1. 試驗/研究之退出與中止：   您可自由決定是否參加本試驗/研究；試驗/研究過程中也可隨時撤銷或中止同意，退出試驗/研究，不需任何理由，且不會引起任何不愉快或影響其日後醫師對您的醫療照顧。為了您的安全，當發生以下情形時，您必須退出試驗/研究：   - 1. 懷孕   2. 任何不舒服之症狀   當試驗/研究執行中有重要的新資訊(指和您的權益相關或是影響您繼續參與意願)，會通知您並進一步說明，請您重新思考是否繼續參加，您可自由決定，不會引起任何不愉快或影響其日後醫師對您的醫療照顧。  計畫主持人亦可能於必要時中止整個試驗/研究之進行。  當您退出本試驗/研究或主持人判斷您不適合繼續參與本試驗/研究時，在退出前已得到的資料將被保留，不會移除。在退出後您可選擇如何處理您先前提供的檢體，與決定是否同意試驗主持人繼續收集您的資料。  1.對我先前所提供的檢體  □我同意繼續授權本試驗/研究使用於本試驗疾病相關的研究。逾越原書面同意使用範圍時，需再次經過我同意。  □不同意繼續授權本試驗/研究使用，但為確保已完成檢查之準確性，同意試驗/研究相關檢體可由實驗室進行再次確認後銷毀。  □不同意繼續授權本試驗/研究使用，請自我退出日起銷毀我之前的本試驗/研究相關檢體。  2.退出後讓試驗主持人繼續收集我的資料，例如經由我的病歷記載取得後續醫療過程、實驗室檢查結果。繼續收集資料期間，仍會維護您的隱私和個人資料的機密性。  □同意收集。  □不同意本試驗/研究繼續收集或檢視我的資料，但可經由公共資料庫查詢之紀錄不在此限。 | |
| 1. 損害補償與保險：   試驗/研究一定有風險，為確保因為參與試驗/研究發生不良反應致造成您的損害時所可能獲得之保障，請您務必詳閱本項說明內容：   - 1. 如依本研究所訂臨床試驗/研究計畫，因發生不良反應造成損害，由國立臺灣大學醫學院附設醫院負補償責任。但本受試者同意書上所記載之可預期不良反應，不予補償。   2. 如依本研究所訂臨床試驗/研究計畫，因而發生不良反應或損害，本醫院願意提供專業醫療照顧及醫療諮詢。您不必負擔治療不良反應或損害之必要醫療費用。   3. 除前二項補償及醫療照顧外，本研究不提供其他形式之補償。若您不願意接受這樣的風險，請勿參加試驗/研究。   4. 您不會因為簽署本同意書，而喪失在法律上的任何權利。 | |
| **(十二)受試者之檢體(含其衍生物)、個人資料之保存、使用與再利用**   - 1. 檢體及剩餘檢體之保存與使用      1. 檢體(含其衍生物)之保存與使用   為研究所需，我們所蒐集您的檢體，將依本研究計畫使用，檢體將保存於國立臺灣大學醫學院附設醫院臨床研究大樓1323實驗室，直至20年保存期限屆滿，我們將依法銷毀。為了保護您的個人隱私，我們將以一個試驗/研究編號來代替您的名字及相關個人資料，以確認您的檢體及與相關資料受到完整保密。如果您對檢體的使用有疑慮，或您有任何想要銷毀檢體的需求，請立即與我們聯絡(聯絡人：施翔蓉醫師，電話：(02)23123456轉261613；聯絡單位：國立臺灣大學醫學院附設醫院，電話：(02)23123456，地址：台北市中正區中山南路七號)，我們即會將您的檢體銷毀。您也可以聯繫本院研究倫理委員會(電話：(02)2312-3456轉263155)，以協助您解決檢體在研究使用上的任何爭議。   - - 1. 剩餘檢體(含其衍生物)之保存與再利用   您的生物檢體將會以專屬號碼進行編碼並在國立臺灣大學醫學院附設醫院的控管下儲存最長20年。所有新的研究計畫都要再經由台大醫院研究倫理委員會審議通過，研究倫理委員會若認定新的研究超出您同意的範圍，將要求我們重新得到您的同意。  是否同意剩餘檢體提供未來地中海型貧血、糖尿病及相關疾病之研究之用，並授權台大醫院研究倫理委員會審議是否需要再取得您的同意：：  □ 1.不同意保存我的剩餘檢體，試驗結束後請銷毀  □ 2.同意以非去連結之方式保存我的剩餘檢體，逾越原同意使用範圍時，需再次得到我的同意才可使用我的檢體進行新的研究     - - 1. 剩餘檢體未來使用之相關資訊   1. 剩餘檢體提供、讓與或授權國內或國外之下列人員使用：   ■目前仍不確定您的檢體是否有可能提供給院外及國外的研究者保管或使用。  如有此種情形，將由本院研究倫理委員會審查檢體使用的適當性，以保障您的權益。   - 1. 剩餘檢體預期利益或預期研究成果：   由於目前還不知道您的剩餘檢體將用於何種醫學研究，因此無法預測可能的研究成果。保存剩餘檢體通常不會對您個人有直接的醫療利益，也不會獲得報酬，但是您所提供檢體，可能促進醫學進步，造福人類健康。   - 1. 剩餘檢體研究與個人疾病相關的檢驗檢查結果   當您的剩餘檢體將來使用於某個研究時，我們將不會通知您研究結果或檢體的檢測結果。一般而言，研究所做的檢測分析，大多還不能運用於醫療照護；但若研究人員認為研究分析的結果，有助於您的醫療照護，也可能會通知您，並協助安排相關檢測與專業諮詢。   - 1. 資料之保存、使用與再利用   在試驗/研究期間，依據計畫類型與您所授權的內容，我們將會蒐集與您有關的病歷資料、醫療紀錄等資料與資訊，並以一個編號來代替您的名字及相關個人資料。前述資料若為紙本型式，將會與本同意書分開存放於研究機構之上鎖櫃中；若為電子方式儲存或建檔以供統計與分析之用，將會存放於設有密碼與適當防毒軟體之專屬電腦內。這些研究資料與資訊將會保存20年。  上述資料與資訊若傳輸至國外分析與統計，您仍會獲得與本國法規相符之保障，計畫主持人與相關團隊將盡力確保您的個人資料獲得妥善保護。試驗結束後，我們可能將試驗資料用於未來醫學研究。   - 1. 與個人疾病相關的檢驗檢查結果   因研究結果尚屬研究階段，不確定性過高/或研究發現不具醫療實用價值，因此我們不會告知您相關的研究檢驗檢查結果。 | |
| **(十三)受試者權益：**   - 1. 如果您在試驗/研究過程中對試驗/研究工作性質產生疑問，對身為患者之權利有意見或懷疑因參與研究而受害時，可與研究倫理委員會聯絡請求諮詢，電話號碼為：(02)2312-3456轉263155。   2. 試驗/研究過程中，與您的健康或是疾病有關，可能影響您繼續接受臨床試驗/研究意願的任何重大發現，都將即時提供給您。如果您決定退出，醫師會安排您繼續接受醫療照護。如果您決定繼續參加試驗/研究，可能需要簽署一份更新版的同意書。   為進行試驗/研究工作，您必須接受施翔蓉醫師、呂金盈醫師、李弘元醫師、吳婉禎醫師、粘峯榕醫師或林家宏醫師的照顧。如果您現在或於試驗/研究期間有任何問題或狀況，請不必客氣，可與在國立臺灣大學附設醫院內科部的施翔蓉醫師聯絡（24小時聯繫電話：0979697907）。   - 1. 本同意書一式2份，試驗主持人或其授權人員已將1份已簽名的同意書交給您，並已完整說明本研究之性質與目的。施翔蓉醫師、呂金盈醫師、李弘元醫師、吳婉禎醫師、粘峯榕醫師或林家宏醫師已回答您有關研究的問題。   2. 參加試驗研究計畫之補助：本研究未提供補助。   3. 若試驗結束後二年內，發現有非預期且直接影響您的安全疑慮，亦將通知您。 | |
| **(十四)本研究預期可能衍生之商業利益及其應用之約定：**  自本試驗/研究取得的資訊可能導致發現、發明或研發商業產品，所有這些權利皆屬於試驗委託者。您與您的家人將不會因這些資訊中的研發成果、發明或其他發現而獲得任何財務利益或金錢補償，或擁有上述發明結果的所有權。 | |
| **(十五)簽名：**  1.試驗主持人、或協同主持人或其授權人員已詳細解釋有關本研究計畫中上述研究方法的性質與目的，及可能產生的危險與利益。  試驗主持人/協同主持人簽名：____________  日期：________年____月____日  在取得同意過程中其他參與解說及討論之研究人員簽名：____________  日期：________年____月____日  2.經由說明後本人已詳細瞭解上述研究方法及可能產生的危險與利益，有關本試驗/研究計畫的疑問，亦獲得詳細解釋。本人同意接受並自願參與本研究，且將持有已簽名的同意書。  受試者簽名： 日期：________年____月____日  出生年月日： ________年____月____日 電話：  國民身分證統一編號： 性別：  通訊地址：  法定代理人/有同意權之人簽名： 日期：________年____月____日  與受試者關係（請圈選）：配偶、父、母、兒、女、其他：_________  出生年月日： ________年____月____日 電話：  國民身分證統一編號：  通訊地址：  *適用醫療法第79條第1項但書或人體研究法第12條第1項但書情形者，其同意權之行使分別依醫療法第79條第2項、人體試驗管理辦法第5條或人體研究法第12條第3、4項規定辦理：  *受試者為**無行為能力者**(未滿七歲之未成年人者或受監護宣告之人)，由法定代理人簽名；受監護宣告之人，由監護人擔任其法定代理人。  * 受試者為**限制行為能力者**(滿七歲以上之未成年人或因精神障礙、其他心智缺陷，致其為意思表示、受意思表示、辨識其意思表示效果之能力，顯有不足，而受法院之輔助宣告者)，應得其本人及法定代理人或輔助人之同意。  * 受試者雖非無行為能力或限制行為能力者，但因**意識混亂或有精神與智能障礙，而無法進行有效溝通和判斷時**，由有同意權之人簽名。有同意權人順序如下：  1.屬新藥、新醫療器材、新醫療技術之人體試驗(人體試驗管理辦法第5條)：  (1)配偶。(2) 父母。(3) 同居之成年子女。(4)與受試者同居之祖父母。(5)與受試者同居之兄弟姊妹。(6) 最近一年有同居事實之其他親屬。  2.屬人體研究（人體研究法第12條）： (1)配偶。(2)成年子女。(3)父母。(4)兄弟姊妹。(5)祖父母。 依前項關係人所為之書面同意，其書面同意，得以一人行之；關係人意思表示不一致時，依前項各款先後定其順序。前項同一順序之人，以親等近者為先，親等同者，以同居親屬為先，無同居親屬者，以年長者為先。  見證人簽名：____________________________日期：________年____月____日  * 受試者、法定代理人或有同意權之人皆無法閱讀時，應由見證人在場參與所有有關受試者同意之討論。並確定受試者、法定代理人或有同意權之人之同意完全出於其自由意願後，應於受試者同意書簽名並載明日期。試驗/研究相關人員不得為見證人。  * 若意識清楚，但無法親自簽具者，得以按指印代替簽名，惟應有見證人。 | |

西元　　　年　月　日病歷委員會審核通過 MR00 -00

西元　　　年　月　日品質暨病人安全委員會審核通過

| **文件編號** | **01400-4-600XXX** | **版次** | **0X** |
| --- | --- | --- | --- |
